# Machine learning and network analysis for diagnosis and prediction in disorders of consciousness

**DOI:** 10.1186/s12911-023-02128-0

**Published:** 2023-02-28

**Authors:** Ajit Narayanan, Wendy L. Magee, Richard J. Siegert

**Affiliations:** 1 Department of Computer Science, School of Engineering, Computer and Mathematical Sciences, Auckland University of Technology, Auckland, New Zealand; 2 Boyer College of Music and Dance, Music Education and Therapy, Temple University, Philadelphia, USA; 3 Department of Psychology and Neuroscience, School of Clinical Sciences, Auckland University of Technology, Auckland, New Zealand

**Keywords:** Machine learning, Music therapy, Disorders of consciousness, Assessment, CRS-R, Network analysis

## Abstract

**Background:**

Prolonged Disorders of Consciousness (PDOC) resulting from severe acquired brain injury can lead to complex disabilities that make diagnosis challenging. The role of machine learning (ML) in diagnosing PDOC states and identifying intervention strategies is relatively under-explored, having focused on predicting mortality and poor outcome. This study aims to: (a) apply ML techniques to predict PDOC diagnostic states from variables obtained from two non-invasive neurobehavior assessment tools; and (b) apply network analysis for guiding possible intervention strategies.

**Methods:**

The Coma Recovery Scale-Revised (CRS-R) is a well-established tool for assessing patients with PDOC. More recently, music has been found to be a useful medium for assessment of coma patients, leading to the standardization of a music-based assessment of awareness: Music Therapy Assessment Tool for Awareness in Disorders of Consciousness (MATADOC). CRS-R and MATADOC data were collected from 74 PDOC patients aged 16–70 years at three specialist centers in the USA, UK and Ireland. The data were analyzed by three ML techniques (neural networks, decision trees and cluster analysis) as well as modelled through system-level network analysis.

**Results:**

PDOC diagnostic state can be predicted to a relatively high level of accuracy that sets a benchmark for future ML analysis using neurobehavioral data only. The outcomes of this study may also have implications for understanding the role of music therapy in interdisciplinary rehabilitation to help patients move from one coma state to another.

**Conclusions:**

This study has shown how ML can derive rules for diagnosis of PDOC with data from two neurobehavioral tools without the need to harvest large clinical and imaging datasets. Network analysis using the measures obtained from these two non-invasive tools provides novel, system-level ways of interpreting possible transitions between PDOC states, leading to possible use in novel, next-generation decision-support systems for PDOC.

**Supplementary Information:**

The online version contains supplementary material available at 10.1186/s12911-023-02128-0.

## Introduction

Severe brain injury can lead to a Prolonged Disorder of Consciousness (PDOC) if it lasts more than 28 days [1]. Awareness (of self, others and the environment) is the central construct of interest in assessment of PDOC due to the medico-legal implications of irreversible coma diagnosis. PDOC patients are categorized as Vegetative State (VS) when there is no evidence of awareness, and Minimally Conscious State (MCS) when there is some discernible evidence of consciousness [2]. Emergence from MCS (EMCS) is associated with increased evidence of awareness, but clear definitions for MCS and EMCS still challenge clinicians [3]. These three categories are accepted as working definitions for which more specific clinical diagnostic markers need to be found.

Improvements in recovery grow steadily with advances in neurological rehabilitation and improved neuroimaging analysis [1]. The management of patients with PDOC continues to challenge clinicians, however, given variability of practice and lack of agreed standards of care for those with PDOC [4]. Therapeutic options and interventions include pharmacological (through intravenous medication) and transcranial magnetic stimulation [5, 6]. Clinical management of patients relies on distinguishing VS from MCS reliably and accurately for improvement of outcomes as well as provision of appropriate clinical care. Long term treatment options remain limited for these complex patients.

Predicting outcomes of PDOC continues to be an active research area. With large-scale studies indicating that only 46% of patients emerge from PDOC after 200 median days [7], there are expectations of PDOC leading to poor outcomes [8]. Historically, prognosis was based on clinical observation and neurological examination of patient behavior to determine evidence of awareness [9]. With improvements in imaging technology, prognosis has been aided by features and abnormalities detected in EEG, CT, PET and MRI images and data [1, 10]. The application of computer models to prognosis and prediction is at a relatively early stage of development, with most focus on predicting mortality through statistical techniques such as regression, and using clinical and imaging data such as event-related potentials (electrophysiological brain responses to a stimulus, e.g. [9, 11]). Such statistical techniques, while providing accurate models based on coefficients and explained variance, may be difficult to interpret for clinical application. More recently, the advantages of using computer models informed by machine learning to help in clinical decision making, in particular, diagnostic support, interpretation of medical images and prediction, are now understood and accepted, as are the potential risks such as excessive trust in their accuracy [12]. Clinical support models need to be interpretable and transparent, however, if the aim is to identify patients most likely to benefit from specialized rehabilitation programs [8]. In short, reliable and interpretable diagnostic markers and outcome predictors for PDOC are still not known [1, 13].

There is growing interest in whether PDOC diagnosis can be based on non-invasive neurobehavioral tests for assessing central nervous system functioning. Measures based on clinician judgements concerning patient responses to markers of consciousness are collected and associated with categories of outcomes. Two such tools for collecting such measures and distinguishing VS from MCS are the Coma Recovery Scale-Revised (CRS-R [14]) and the MATADOC (Music Therapy Assessment Tool for Awareness in Disorders of Consciousness [15]).

The CRS-R is a well-established tool for detecting changes in the neurobehavioral status of PDOC using six subscales assessing auditory, visual, motor, oromotor, communication and arousal responsiveness, with the lowest scores representing absent or reflexive activity and the highest behaviors mediated by cognitive input [14]. Auditory measures involve auditory startle, localization of sound and movement to language command, with criteria provided for identifying responses for different methods of measurement. CRS-R scores, together with clinical information, have been demonstrated through statistical univariate and multivariate analysis to be predictors of functional improvement [16]. Also, CRS-R scores with additional clinical information have been demonstrated through binary logistic regression to predict favourable versus unfavourable outcome, but with only 54% of the variance explained in the model [17].

Music interventions have received closer examination in recent years as a neurobehavioral assessment and treatment option for people with PDOC. The development of the MATADOC is based on music as a non-language stimulus for assessment of awareness which, unlike CRS-R, does not rely on patient ability to understand and respond to language on certain items [15]. The MATADOC uses 14 items for rating responses in the five behavioral domains of auditory, visual, motor, communication and arousal. A preliminary study of concurrent validity of MATADOC repeated measures with the CRS-R identified significant correlations on diagnostic outcomes and for visual, auditory, communication and arousal subscales [18], indicating that both tools may contain useful information to help with diagnosis and prognosis. More detail on these two tools is provided under Methods.

The advantage in using neurobehavioral measures for diagnosis includes greater focus on a limited number of visual, auditory, verbal, motor and arousal constructs without needing to harvest data from other sources. The measures are easily applicable without the need for complex scanning and imaging equipment. Also, the measures are behaviorally interpretable. However, it is not known whether there is sufficient information in such neurobehavioral measures for computer-aided predictive diagnosis and prognosis for use in clinical decision support systems.

The aim of this study is to explore whether machine learning can use neurobehavioral data for diagnostic assessment and prediction as well as provide reasons for its decisions. Also, there is very little understanding of how neurobehavioral measures can be used for possible intervention strategies to help patients move from VS to MCS. Recent developments in network analysis in other areas of clinical healthcare (e.g. mental disorders [19], depression [20, 21]) indicate that it may be possible to provide system-level models of interactions between measures for different categories of patients. Differences in network topology between different diagnostic groups and various metrics associated with network analysis may provide possible intervention pathways for future transition investigations. In particular, it is not currently known whether the MATADOC and the CRS-R can be used together to reach consensus of agreement on diagnostic outcomes for use in decision making. Nor is it known whether the MATADOC and the CRS-R variables can be used together to identify reasons for diagnosis as well as provide potential pathways for intervention in the clinical management of PDOC patients.

The study can be broken down into four stages. The first stage (S1) assesses the quality and reliability of neurobehavioural items, scales and sample numbers for subsequent analysis. The second stage (S2) checks on the level of diagnostic agreement between the two neurobehavioral tools to determine whether they can be used reliably in ML or whether additional, induced diagnostic categories need to be derived. The third stage (S3) applies ML methods for data fitting and prediction. The final stage (S4) is to generate system level views of coma states. Further information on all techniques is supplied below and in the Additional file 1), and comparisons of results with previous, relevant ML research are presented in the “Discussion” section.

## Materials and methods

### Patients

The sample consists of 74 PDOC patients aged 16–70 years with PDOC recruited from three centers in the USA, UK and Ireland which have specialist programs for the assessment and treatment of people with DoC. Participants were medically stable inpatients with a PDOC that had persisted longer than 4 weeks and whose awareness was under active investigation at the time of recruitment to the study. Exclusion criteria were: nonfluent in English, known pre-morbid hearing impairment, known musicogenic epilepsy, or suspected Locked-in Syndrome. This study was approved by research ethics boards of all participating institutions. Consent was gained from patients’ legally authorized representatives.

### Procedures

MATADOC and CRS-R data were collected concurrently for each patient on four occasions over two weeks by experienced clinician assessors blinded to the other measure’s outcomes on each occasion. The MATADOC consists of 14 items forming three subscales. The first subscale (Principal) consists of 5 items assessing: responses to visual stimuli, responses to auditory stimuli, awareness of musical stimuli, responses to verbal commands, and arousal. The second subscale has two items: behavioral response to music, and musical response. The third subscale has the remaining seven items and assesses motor, communication and emotional behaviors for use in rehabilitation programs, and are not used in this study. MATADOC protocols elicit behavioral responsiveness on each occasion using live music during contacts of 15–30 min duration. The MATADOC data for this study therefore consists, for each occasion, of the five Principal subscale items, the two secondary scale items, plus a coma diagnostic state for that occasion calculated (in accordance with the measurement protocol) by summing the five Principal items (individual item scores 0, 1 and 2 for VS, MCS and EMCS, respectively; range 0–3 VS, 4–7 MCS, 8–10 EMCS). See [15] for further details of MATADOC and its protocols.

The CRS-R consists of 6 subscales (function scales, FS): auditory, visual, motor, oromotor (verbal), communication, and arousal. Each FS is scored (between 0–3 and 0–6) where the lowest score represents reflexive activity and the highest cognitively-mediated behaviors. Each FS has thresholds for diagnostic state outcome, calculated as follows from ranges of values: Auditory (≤ 2 VS; 3–4 MCS); Visual (≤ 1 VS; 2–5 MCS); Motor (≤ 2 VS; 3–5 MCS; 6 EMCS); Oromotor (≤ 2 VS; 3 MCS); Communication (0 VS; 1 MCS; 2 EMCS). The summed score across the six subscales varies from 0 to 23, with a score of 0–9 signifying VS and 10 or more signifying MCS or EMCS. The CRS-R protocol delivers tasks in the motor, visual, auditory and communication domains in contacts of 15–30 min duration. In summary, the CRS-R data consist of these 6 subscale scores, plus an overall diagnostic state calculated from the summed scale score. Each patient has four such sets of data for the four different occasions. See [14] for further details of CRS-R and its protocols. Additionally, gender, age, time since onset and primary cause of injury were recorded for each patient. All data were anonymized for analysis.

### Data preparation

Categories VS, MCS and EMCS were coded 0, 1 and 2 for MATADOC items and treated as ordinal. CRS-R subscale scores were treated as scale. The items and scales were averaged across the four occasions to control for variability of raters (different raters were used in different centers and by occasion). The overall diagnostic outcome for each patient was calculated using the five Principal subscale items of MATADOC and the six function subscales of CRS-R after the fourth and final occasion. This resulted in each of the patients having 7 averaged MATADOC items over the four occasions, 6 averaged CRS-R scale values averaged over the four occasions, one MATADOC diagnostic outcome calculated from the final MATADOC occasion and one overall CRS-R outcome from the final CRS-R occasion (15 measures in total, 13 of which are neurobehavioral measures and two of which are calculated diagnostic outcomes). Also, patient demographics such as age at time since onset and gender, as well as primary cause of injury (PCOI), were included in the dataset as possible factors. The 13 averaged MATADOC items and CRS-R functional scales are referred to as ‘variables’, and the two final occasion outcomes are referred to as ‘calculated diagnostic outcomes’ in the analysis below.

### Statistical methods (with reference to Stages S1–S4)

S1:McDonald’s omega (ω) and factoring are used to test the reliability of the 13 variables for internal consistency and scale formation to check for use in subsequent analysis. Also, gender, age and PCOI are tested for effects on total scale scores. Post-hoc power analysis is used to demonstrate sufficient cases for subsequent analysis.

S2: Calculated diagnostic outcomes from MATADOC and CRS-R are compared by Fleiss’ kappa (κ) to identify level of agreement between the two instruments for supervised learning. If the agreement is not high, unsupervised techniques can be used to generate additional, induced categories based on the shared patterns of responses to MATADOC and CRS-R variables, guided by the original, calculated diagnostic outcomes (more details in S3 below).

S3: Artificial neural networks and decision trees are used for supervised learning, and cluster analysis for unsupervised learning. Supervised learning in this study uses the calculated diagnostic outcomes to train models to produce the most accurate classification. Unsupervised learning finds patterns of variable values to identify induced categories and is used here due to low agreement on the original, calculated diagnostic outcomes (Results below). Artificial neural networks in the form of perceptrons and decision trees in the form of J48 [22] are used for supervised learning using tenfold cross-validation. ANNs are structured networks with input nodes and output nodes that learn relationships between attribute data and class data by weighting input attribute data in such a way that specific and desired class outputs are produced. The weights are learned by repeated presentation of the data (feedforward) with adjustments made to those weight (backpropagation) so that the desired class outcomes are more likely to be produced with subsequent presentations of data. Such gradual refinement of weights through repeated presentations of the data, together with additional layers of weights through ‘hidden’ layers of nodes, can lead to ANN models (perceptrons) that can successfully classify data containing complex non-linear relationships. In this study, one additional layer of nodes was found to be adequate for successful modelling. Interpreting ANN weights and the network model can be problematic for decision support purposes, since weights are mathematical values and data may be normalized or otherwise transformed prior to input to keep weights within specific ranges as well as help the ANN converge to an appropriate set of weights. Decision trees on the other hand produce tree representations where internal nodes of the tree represent data attributes and leaf nodes represent class values. Each branch from an attribute represents a decision based on the effectiveness of splitting the attribute’s data at a certain point for capturing cases of a specific class outcome. A path from the root node to the leaf node represents a series of decisions based on attribute values leading to that class outcome. No normalization or scaling of data is required, leading to an interpretable classification model that is easy to explain. In this study, both ANNs and decision trees are used for comparative purposes as well as to complement each other so that effective and interpretable models are produced for further evaluation and discussion with regard to coma decision support. J48 is tree-generating algorithm based on a top-down strategy of finding, at each level of the tree, the attribute or attributes with the most information for splitting the samples into the specified class values using information theoretic concepts of entropy. Intuitively, the top-most node in the decision tree is the most important attribute in terms of information gain based on class values, and nodes further down the tree are increasingly less important in terms of contributing information to classification. The data are divided into increasingly smaller subsets until all samples at a leaf node fall in the same class. Since finding a tree where every leaf contains samples of the same class can lead to large trees that are difficult to interpret (‘overfitting’), pruning can be used in J48 to remove branches with small numbers of samples so that the largest tree with the most generalizability is returned. Pruning requires an acceptance of classification error at the leaf nodes (confidence factor) and the setting of a minimum number of samples to be classified at a leaf node.

Cross-validation models present their prediction results in confusion matrices, where rows represent actual cases falling into diagnostic outcomes VS, MCS and EMCS, and columns their predicted counterparts. Three metrics are generated from confusion matrices: true positive rate (TPR), precision (P) and receiver operating characteristic area under the curve (ROC AUC: calculated by plotting TPR against false positive rate FPR at various threshold settings). Unsupervised learning takes place through k-means clustering, where samples in one cluster should have more in common with each other than they have in common with samples in another cluster. The number of clusters to be found is set by k, which is 3 in our study, with the intention of finding induced VS, MCS and EMCS categories through shared data patterns among the variables. Supervised machine learning is then repeated using the three induced clusters as diagnostic categories and the results compared with those obtained with the calculated diagnostic outcomes. To identify differences in ML metrics and compare ML performance, paired T-tests are used, where the metrics (TPR, P, ROC AUC) for the calculated diagnostic outcomes and induced categories are treated as a column of subjects (9 rows) and compared against each other (column by column) across different experimental conditions. The use of T-tests adds a degree of formality and rigor to such comparisons and provides some assurance that differences may not be due to chance. Additional file 1 (Machine Learning) provides further details of these metrics as well as the ML parameters used for the ANNs, J48 and k-means clustering. Many on-line sources of information and courses are available on these methods as well as introductory books with applications to healthcare (e.g. [23]).

S4: Network analysis is a rapidly growing area in health science aimed at identifying structural relations among variables through graphical representations, where nodes represent variables such as attitudes, cognitive states, symptoms and behaviors, and links (edges) represent a statistical relationship, usually correlation or other forms of association measures, that convey information about the strength or direction of the relationship [24]. Partial correlations are used for edge calculation, where correlations between two variables are calculated after removing the effects of all other variables. Spring layout (a force-directed graph drawing algorithm [25] is adopted for network visualization, where edges are regarded as springs pulling nodes together. Larger edge weights (partial correlations) imply a stronger inter-node attractive force. Centrality measures [26] provide further information on network nodes, such as ‘betweenness’ (how well a node acts as a connecting point based on the number of paths through that node to other nodes), ‘closeness’ (how close a node is to other nodes using the average weight of the paths from that node) and ‘degree’ (the sum of all absolute weights from that node, or strength). Centrality measures are presented as standardized z scores (the number of standard deviations by which a value is above or below the mean) to allow comparison across network variables.

All analysis was carried out with SPSS Versions 27 and 28 (statistical analysis, ANNs, k-means clustering), WEKA Version 3.8.4 (ANNs, k-means clustering, J48) and JASP Version 0.9.2 (statistical analysis, network analysis).

## Results

### Overview of data

The median age of the 74 patients was 38.3 years old (range 16–75, quartiles 25 and 57), and the average time since onset was 20.5 months (1–216, 4, 21) with median 6. There were 22 female and 52 male patients. For PCOI, trauma was recorded for 35 patients, anoxia/hypoxia for 24, stroke for 8, subarachnoid hemorrhage for 5 and hypoglycemia for 2. Table 1 gives an overview of all 13 variables after averaging across the four measured timepoints, together with the shortened versions of variable names as used in the remainder of this study.

**Table 1:** Overview of 13 averaged variables (MATADOC items 1–7, M-prefix; CRS-R subscales 1–6, C-prefix), with shortened abbreviations AM1-AC6 (for Averaged MATADOC item 1—averaged CRS-R subscale 6) under the Item column, n = 74

Item	Minimum	Maximum	Mean		SD	Variance	Skewness		Kurtosis	
	Statistic	Statistic	Statistic	Std. Error	Statistic	Statistic	Statistic	Std. Error	Statistic	Std. Error
MVisual (AM1)	0.00	2.00	0.96	0.08	0.72	0.51	0.06	0.28	− 1.28	0.55
MAuditory (AM2)	0.00	2.00	1.03	0.07	0.58	0.33	− 0.07	0.28	− 0.66	0.55
MAwareness (AM3)	0.00	2.00	0.77	0.06	0.48	0.23	0.03	0.28	− 0.29	0.55
MVerbal (AM4)	0.00	2.00	0.63	0.07	0.64	0.41	0.70	0.28	− 0.69	0.55
MArousal (AM5)	0.25	2.00	1.43	0.05	0.42	0.18	− 0.10	0.28	− 0.82	0.55
MBehaviour (AM6)	6.00	12.00	8.66	0.18	1.51	2.27	0.29	0.28	− 0.66	0.55
MMusical response (AM7)	7.00	13.25	8.81	0.21	1.80	3.23	1.00	0.28	− 0.09	0.55
CAuditory (AC1)	0.00	4.00	1.66	0.13	1.13	1.27	0.71	0.28	− 0.67	0.55
CVisual (AC2)	0.00	5.00	1.79	0.19	1.62	2.64	0.46	0.28	− 1.02	0.55
CMotor (AC3)	0.00	6.00	2.68	0.23	1.97	3.87	0.30	0.28	− 1.46	0.55
COro-motor (AC4)	0.00	3.00	1.34	0.08	0.73	0.53	0.42	0.28	− 0.21	0.55
CCommunication (AC5)	0.00	2.00	0.19	0.05	0.43	0.18	2.48	0.28	5.80	0.55
CArousal (AC6)	0.00	3.00	1.69	0.06	0.56	0.31	0.25	0.28	0.75	0.55
Averages	1.10	4.60	2.50	0.12	1.00	1.30	0.52	0.28	− 0.17	0.55

### S1: reliability and scale structure

McDonald’s ω was 0.93 (95% CI lower and upper bounds 0.90 and 0.95) and 0.88 (0.83, 0.93), respectively, for MATADOC (mean 22.28, Std Dev 5.25) and CRS-R (9.36, 5.27) variables when formed into separate scales. The mean inter-item correlation within the MATADOC was 0.632 and within the CRS-R 0.549. Combining MATADOC and CRS-R scales into one scale resulted in 0.93 ω (0.91, 0.95), with average inter-item correlation of 0.56. Factor analysis (principal component analysis) identified two factors with eigenvalues greater than 1 for the combined scale and factor loadings that clearly separated MATADOC and CRS-R items, indicating that the combined scale was not unidimensional. Hence, the two scales were kept separate for subsequent analysis below. Post-hoc power calculations using scale means and standard deviations indicated > 98% power (Type 1 error rate α = 0.05) for distinguishing VS, MCS and EMCS.

### S2: diagnostic outcomes in MATADOC and CRS-R

**Fig. 1:**
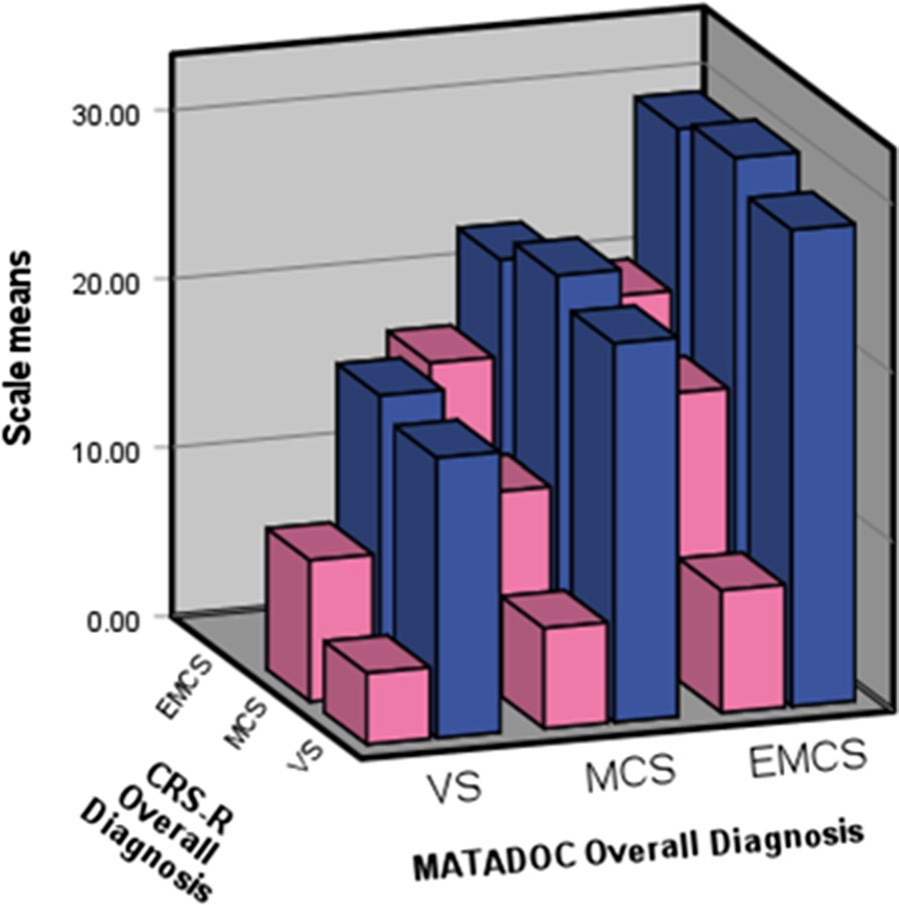
MATADOC scale (blue) and CRS-R scale (pink) means (y axis) by MATADOC (x axis) and CRS-R (z axis) diagnostic outcomes VS, MCS and EMCS calculated from the five items making up the MATADOC Principal subscale and six CRS-R function sub-scales

For MATADOC, the number of VS (Vegetative State) cases as calculated from the five Principal scale scores on the last occasion was 23 (31.1%), MCS (Minimally Conscious State) 39 (52.7%) and EMCS (Emergence from MCS) 12 (16.2%). The corresponding figures for CRS-R were 35 VS (47.3%), 33 MCS (44.6%) and 6 EMCS (8.1%). The two scales showed significant differences (*p* ≤ 0.001) in mean value through analysis of variance by MATADOC and CRS-R calculated diagnostic outcomes, with means scale scores rising from VS to MCS and EMCS (Fig. 1). A breakdown by means for diagnostic categories for all individual variables and scales is given in Additional file 2.

Fleiss’ κ of inter-rater agreement between MATADCOC and CRS-R calculated diagnostic outcomes was 0.39 (*p* ≤ 0.01, 95% lower and upper confidence intervals 0.21 and 0.56, respectively), indicating only fair agreement. Table 2 shows the levels of agreement and disagreement for overall diagnosis, with 63.6% of cases (47/74) having the same agreed outcome across VS (κ = 0.55), MCS (κ = 0.30) and EMCS (κ = 0.24), as shown by the sum of leading diagonal figures. Two MATADOC VS cases were diagnosed as MCS by CRS-R, and 13 CRS-R VS cases were diagnosed as MCS by MATADOC. Given only fair agreement in calculated diagnostic outcomes, inducing new diagnostic categories from shared patterns of data through unsupervised ML for further exploration in supervised ML can be justified for additional ML exploration (further details below).

**Table 2:**
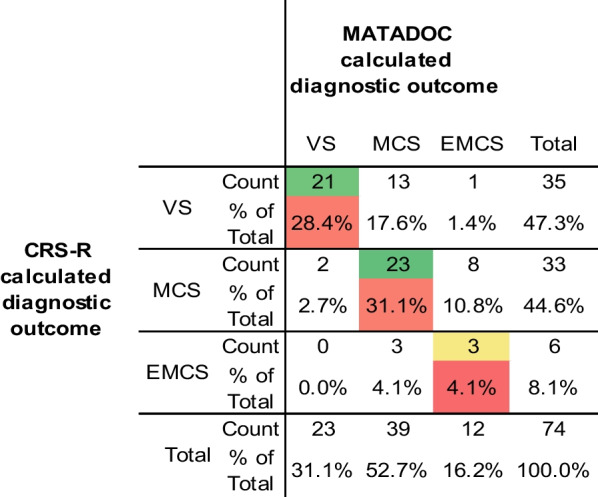
Overall calculated diagnostic outcome agreement between MATADOC and CRS-R

The lead diagonal gives the agreement between MATADOC (columns) and CRS-R (rows) outcomes, with all other entries signifying type of disagreement. Total cases in each diagnostic category are provided at the end of rows and columns for each of the two tools

*VS* vegetative state, *MCS* minimally conscious state, *EMCS* emergence from MCS

### S3: supervised ML

A multilayer perceptron (MLP) with 13 input variables (from the two tools), 7 hidden units, six output units (three for each calculated MATADOC diagnostic outcome state, three for each calculated CRS-R outcome state), 0.1 learning rate, 0.1 momentum and 10,000 epochs was found to perfectly fit all MATADOC and CRS-R cases. This architecture was then used for tenfold cross-validation. 59 of 74 cases were correctly predicted for MATADOC diagnostic outcomes using the 13 variables (Table 3(a), top left, leading diagonal), resulting in 0.80 overall TPR (Table 4(a), top table, upper left, TPR average). The corresponding results for CRS-R diagnostic outcomes were 58 of 74 cases and 0.78 (Table 3(a), top table, upper middle, leading diagonal; Table 4(a), top table, upper right, TPR average). The differences in metrics for TPR, P and ROC AUC for MATADOC prediction and CRS-R prediction were significant (*p* ≤ 0.05), with MATADOC having higher values than CRS-R. When primary cause of injury (PCOI) was added as a factor, the number of correctly predicted cases fell to 57 cases for MATADOC and 55 cases for CRS-R outcomes respectively (Table 3(a), middle left and right, leading diagonals), but still with significant differences (*p* ≤ 0.01) between the two sets of metrics (Table 4(a), middle table). Diagnostic accuracy for VS, in particular, improved to 0.96 TPR for MATADOC (from 0.87), and 0.86 from 0.83 for CRS-R (Table 4(a), middle left and right, VS row). Adding other factors (Age, Time since onset, Gender) did not improve accuracy of the MLP.

**Table 3:**
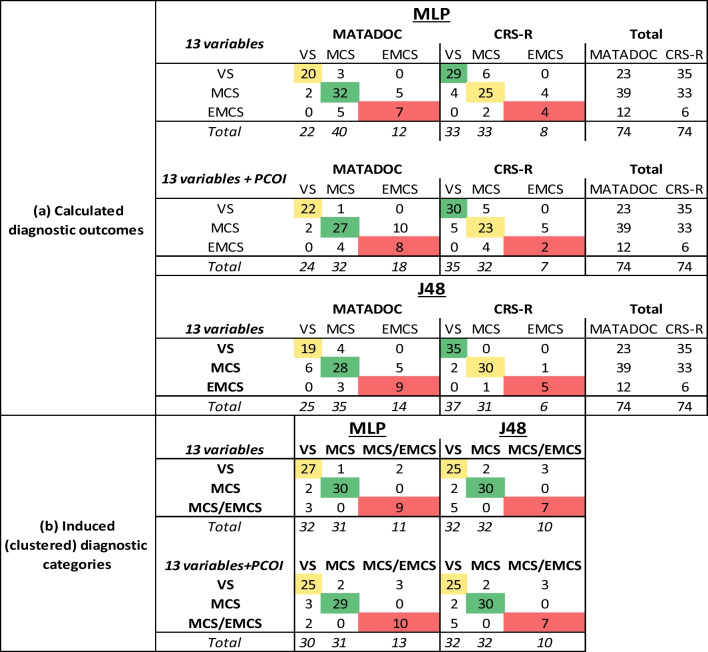
Confusion matrices for multilayer perceptron (MLP) and J48 using tenfold cross-validation, with columns signifying predictions and rows specifying actual cases

The upper half (a) contains figures for the calculated MATADOC and CRS-R diagnostic outcomes using the 13 variables without and with primary cause of injury (PCOI) as a factor, and the lower half (b) for the three induced diagnostic categories without and with PCOI. The lead diagonals (in color) identify correct predictions

**Table 4:**
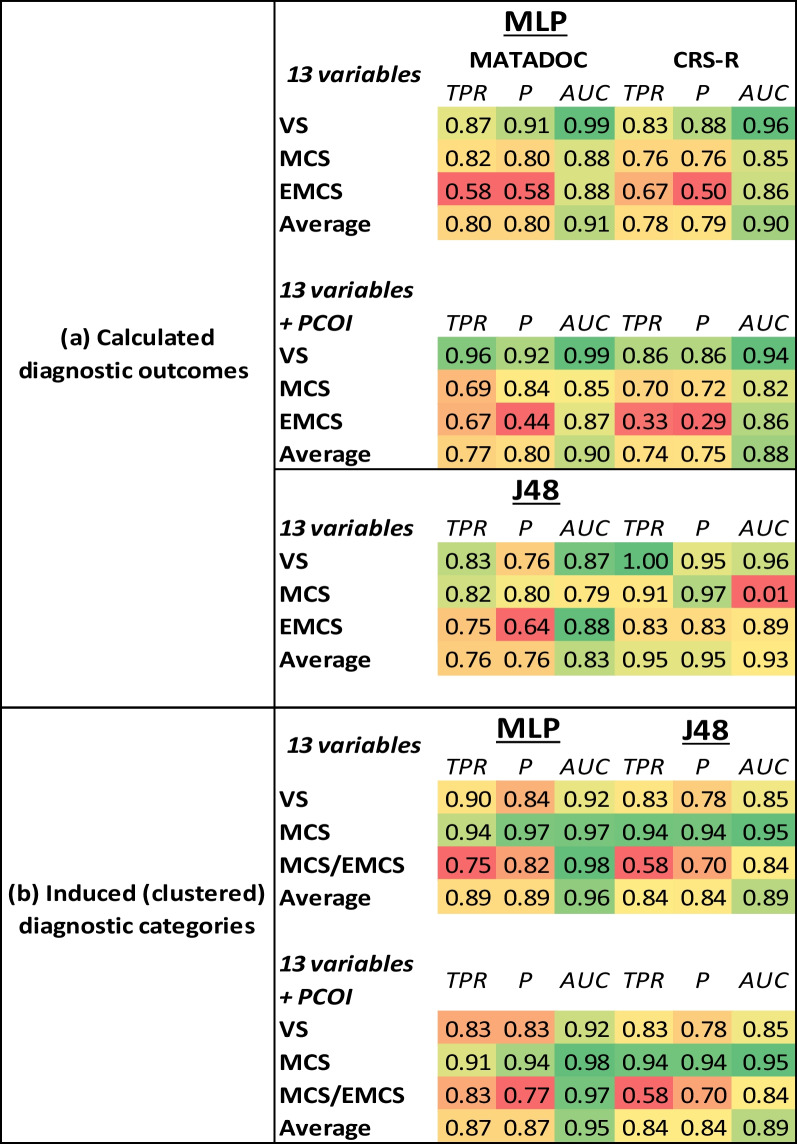
Three metrics [True Positive Rate (TPR), Precision (P) and AUC ROC] used in MLP and J48 cross-validation for the (a) calculated MATADOC and CRS-R diagnostic outcomes, and (b) induced diagnostic categories, without and with PCOI included as a factor

Values range from green (high) to red (low)

Moving to J48 as the learning model, tenfold cross-validation without PCOI led to 56 cases being correctly classified for MATADOC diagnostic outcome and 70 for CRS-R diagnostic outcome (Table 3(a), bottom left and middle, leading diagonals) with significant differences (*p* ≤ 0.01) in metrics. This time, CRS-R outcomes had a higher TPR value (0.95) than MATADOC (0.76) (Table 4(a), bottom left and right, average TPR columns). Including PCOI had no effect on J48 predictions, nor did any other factor.

**Fig. 2:**
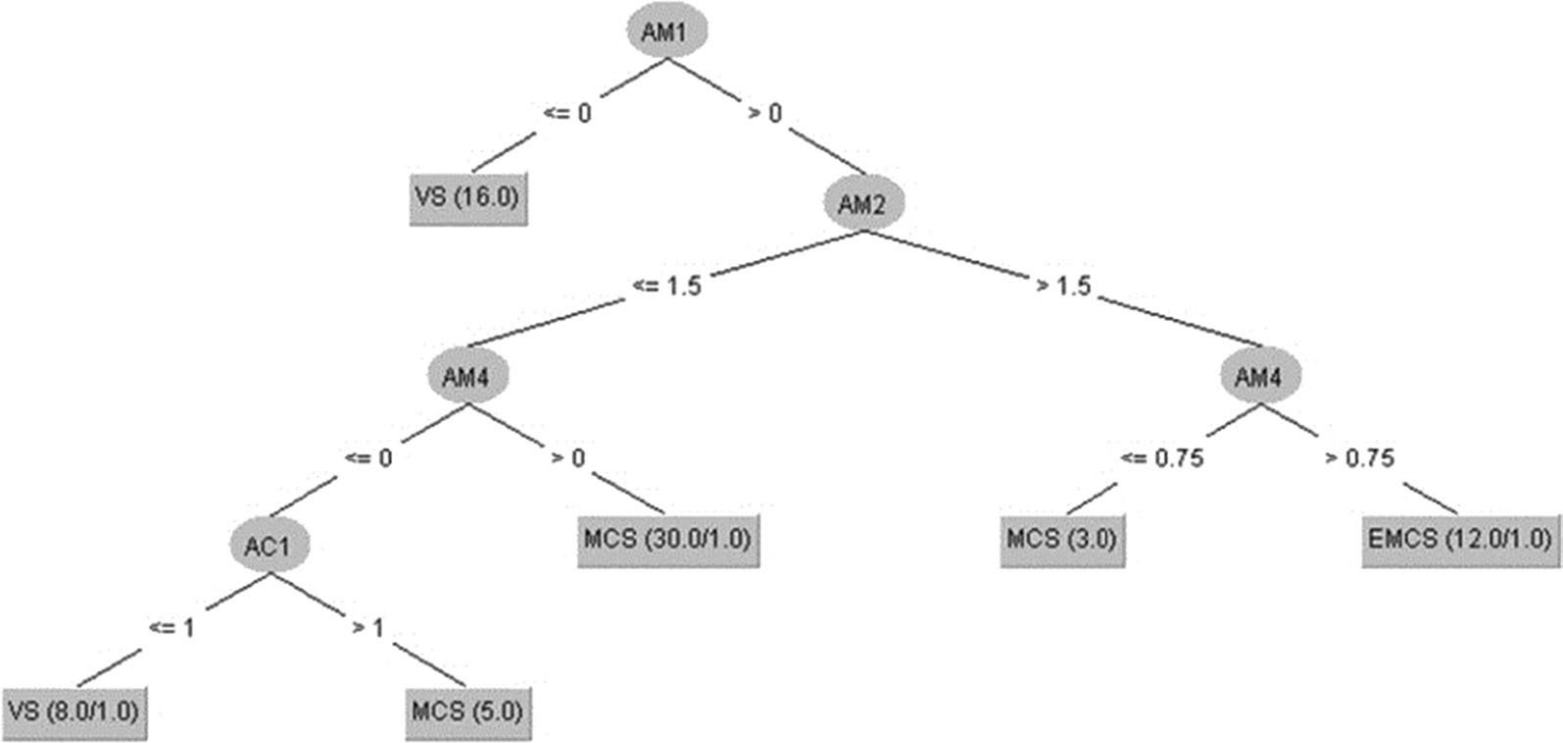
J48 data-fitting decision tree for all 74 samples, with cases represented by MATADOC diagnostic outcomes located at the leaves of the tree, and (X,Y) meaning X cases correctly captured including Y, if present, false positives. See Table 1 for Key to AM1-7 and AC1-6. Values “ <  = 0” are to be interpreted as “ = 0”, and all other relations are normal (e.g. ‘ > ’ = greater than, etc.)

**Fig. 3:**
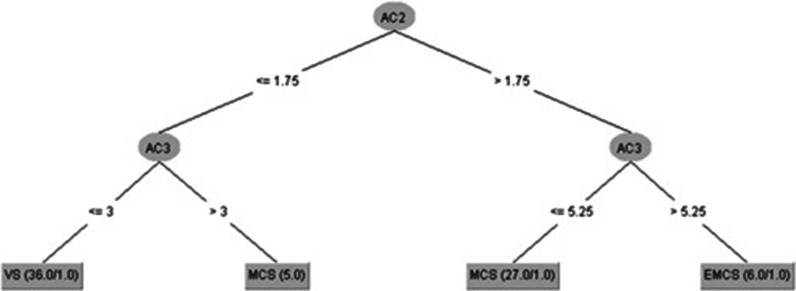
J48 data-fitting decision tree for all 74 samples, with cases represented by CRS-R calculated diagnostic outcomes located at the leaves of the tree, and (X,Y) meaning X cases correctly captured including Y, if present, false positives. See Table 1 for Key to AM107 and AC1-6

Model-fitting (no withheld cases) using all 13 variables from both measures with J48 produced a decision tree correctly locating 71 (96%) of the 74 samples for each of the MATADOC (Fig. 2) and CRS-R (Fig. 3) calculated diagnostic outcomes. One CRS-R variable (Auditory) was used in the tree for MATADOC outcomes (Fig. 2), and only CRS-R variables were needed for CRS-R outcome (Fig. 3). When paths from the root of the tree to the leaves are traced out as rules, the following full rule set (RS1) captures the information in Fig. 2 (MATADOC calculated diagnostic outcome, with AM1 = Mvisual, AM2 = Mauditory, AM4 = Mverbal, AC1 = I,VS = Vegetative State, MCS = Minimally Conscious State, EMCS = Emergence from MCS):

- ***RS1:***
- ***If AM1 = 0 then MATADOC VS (16 cases correctly classified, including 0 false positives)***
- ***If AM1 > 0 and AM2 ≤ 1.5 and AM4 = 0 and AC1 ≤ 1 then MATADOC VS (8, 1)***
- ***If AM1 > 0 and AM2 ≤ 1.5 and AM4 = 0 and AC1 > 1 then MATADOC MCS (5, 0)***
- ***If AM1 > 0 and AM2 ≤ 1.5 and AM4 > 0 then MATADOC MCS (30, 1)***
- ***If AM1 > 0 and AM2 > 1.5 and AM4 ≤ 0.75 then then MATADOC MCS (3, 0)***
- ***If AM1 > 0 and AM2 > 1.5 and AM4 > 0.75 then then MATADOC EMCS (12, 1)***

Similarly, the rules (RS2) for Fig. 3 (CRS-R calculated diagnostic outcome) are (AC2 = Cvisual, AC3 = Cmotor):

- ***RS2:***
- ***If AC2 ≤ 1.75 and AC3 ≤ 3 Then CRS-R VS (36, 1)***
- ***If AC2 ≤ 1.75 and AC3 > 3 Then CRS-R MCS (5, 0)***
- ***If AC2 > 1.75 and AC3 ≤ 5.25 Then CRS-R MSC (27, 1)***
- ***If AC2 > 1.75 and AC3 > 5.25 Then CRS-R EMCS (6, 1)***

### S3: unsupervised ML

K-means clustering on all 13 variables plus calculated MATADOC and CRS-R outcomes resulted in 30 VS cases as diagnosed by MATADOC and CRS-R falling in cluster 1, 32 MCS cases as diagnosed by MATADOC and CRS-R falling in cluster 2, and a mixture of 12 cases of EMCS from MATADOC outcomes and MCS from CRS-R outcomes in cluster 3. Univariate tests confirmed the statistical significance of each clustering variable, in line with the earlier reported statistically significant ANOVA results. Clusters 1, 2 and 3 are labelled ‘induced diagnostic categories’ for the analysis below to separate them from the original, calculated diagnostic outcomes.

Repeating supervised learning (tenfold cross-validation) with the same MLP architecture as before but this time using the clusters as new, induced categories resulted in 66 cases (89%) being correctly classified with the MLP and 62 cases (84%) with J48 without PCOI (Table 3(b), upper half, left and right). Adding PCOI did not increase the number of true positives for MLP and J48 (Table 3(b), lower half, left and right). The improvements in metrics for MLP between the calculated diagnostic outcomes of MATADOC and CRS-R on the one hand (Table 4(a)), and induced diagnostic categories on the other (Table 4(b)), without and with PCOI (upper and lower), were significant (*p* ≤ 0.01), with the metrics for induced categories having higher values. There were no significant differences between the calculated MATADOC and CRS-R outcomes on the one hand, and induced categories on the other, for J48.

Independent variable importance using a data-fit MLP for maximum knowledge extraction identified Mbehavior (importance = 0.162, normalized importance = 100% without PCOI; 0.172, 100% with PCOI), Mmusical Response (0.155, 95.4%; 0.143, 83.2%) and Cmotor (0.15, 92.6%; 0.153, 88.8%) as the most important variables for induced categories, without and with PCOI (Table 5).

**Table 5:**
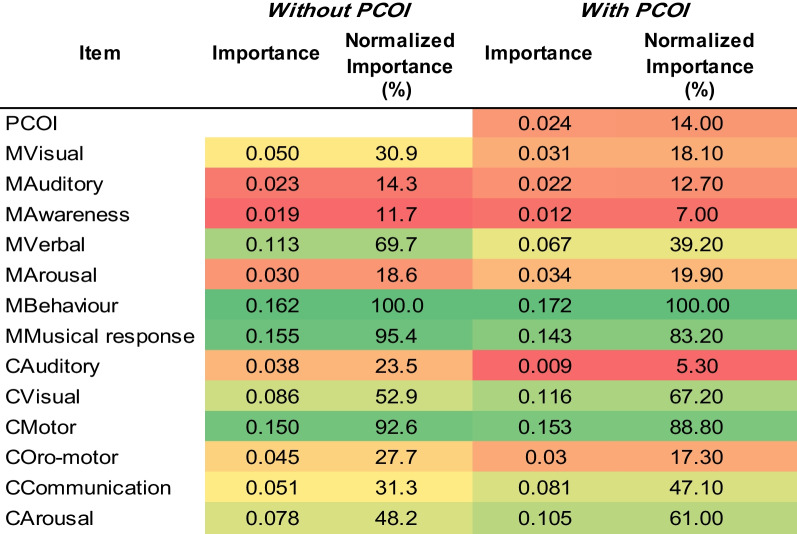
List of most important variables for data-fitting using the MLP and the three clusters as induced categories, without PCOI (left two columns) and with PCOI (right two columns). (See Additional file 1 for details of sensitivity analysis and importance.)

A data-fit model using the induced categories with J48 was 99% accurate with and without PCOI (one VS case wrongly classed with MCS) and produced a decision tree and set of rules (RS3) involving Cmotor (AC3), Mverbal (AM4) and Mmusical response (AM7):

- ***RS3:***
- ***If AC3 ≤ 2 and AM7 ≤ 8.5 Then Cluster MCS (31,0)***
- ***If AC3 ≤ 2 and AM7 > 8.5 Then Cluster VS (11,1)***
- ***If AC3 > 2 and AM7 ≤ 9.25 Then Cluster VS (17.0)***
- ***If AC3 > 2 and AM7 > 9.25 and AM4 ≤ 0.75 Then Cluster VS (3,0)***
- ***If AC3 > 2 and AM7 > 9.25 and AM4 > 0.75 Then Cluster MCS/EMCS (12,0)***

### S4: Network analysis

**Fig. 4:**
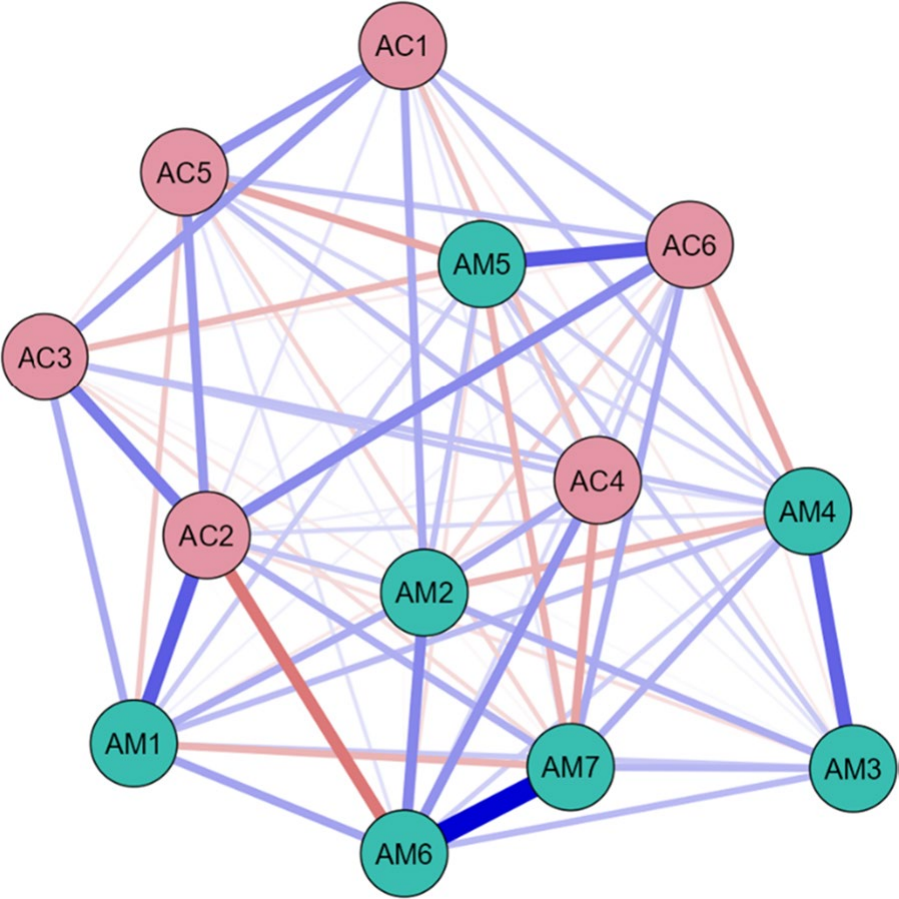
Partial correlation network of all 13 variables, irrespective of original outcome or induced category of patient (n = 74), showing relationships between MATADOC (AM) variables and CRS-R (AC) variables, with blue signifying positive relationships and red negative. Thickness of line indicates strength of relationship. See Table 1 for key to variable names

Network analysis of the 13 variables revealed the underlying relationships irrespective of any outcome (Fig. 4), with MATADOC and CRS-R variables closely located to each other. Strong positive within-scale relationships are identified between Mawareness (M3) and Mverbal (M4), and between Mbehavior (M6) and Mmusical response (M7). Similarly, strong positive within-scale relationships exist between Cvisual (C2) and Cmotor (C3). Strong positive relationships across scales are identified between Mvisual (M1) and Cvisual (C2), and between Marousal (M5) and Carousal (C6).

**Fig. 5:**
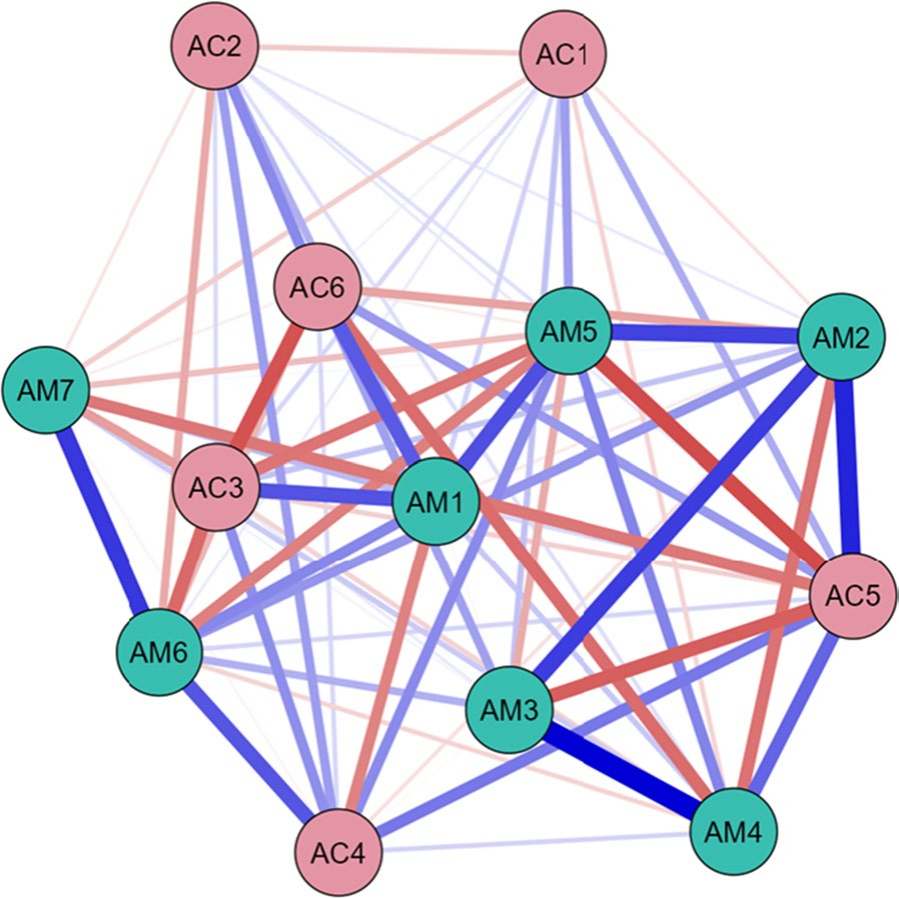
Partial correlation network of all 13 variables for induced category VS only (N = 30), showing relationships between MATADOC (AM) variables and CRS-R (AC) variables, with blue signifying positive relationships and red negative. Thickness of line indicates strength of relationship. See Table 1 for key to variable names

**Fig. 6:**
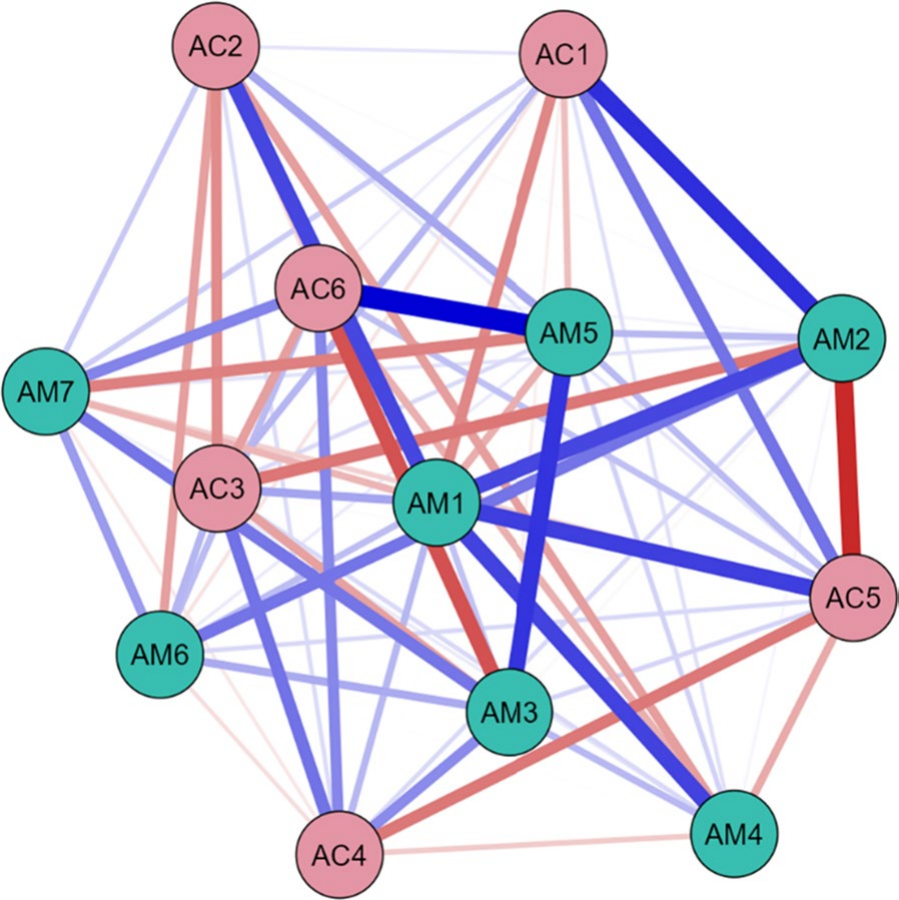
Partial correlation network of all 13 variables for induced category MCS only (N = 32), showing relationships between MATADOC (AM) variables and CRS-R (AC) variables, with blue signifying positive relationships and red negative. See Table 1 for key to variable names

**Fig. 7:**
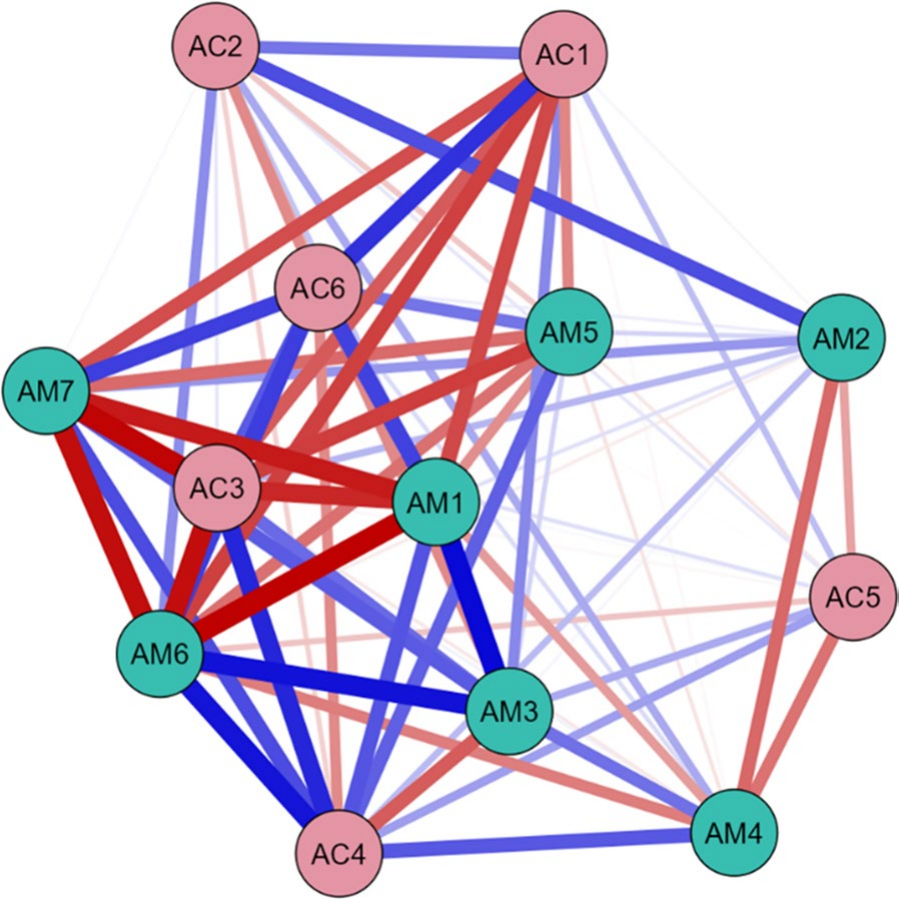
Partial correlation network of all 13 variables for induced category MCS/EMCS only (N = 12), showing relationships between MATADOC (AM) variables and CRS-R (AC) variables, with blue signifying positive relationships and red negative. See Table 1 for key to variable names

**Fig. 8:**
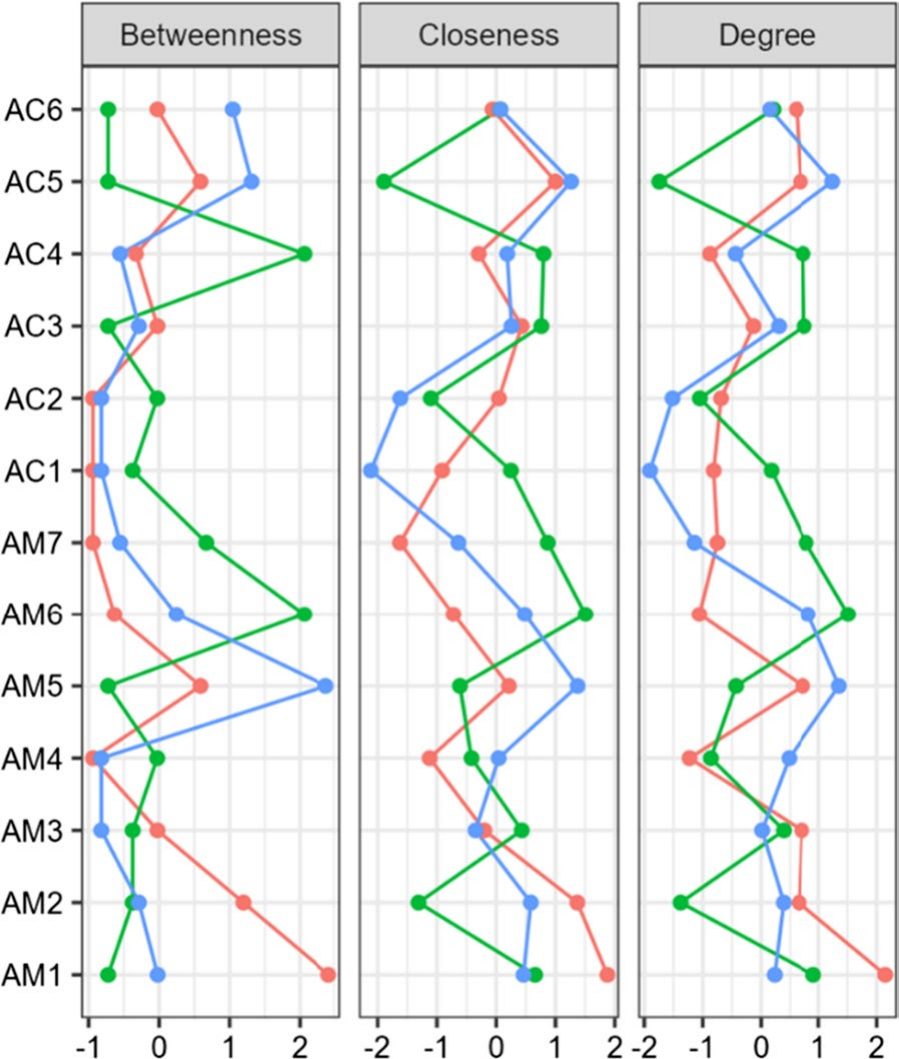
Centrality plots of Betweenness, Closeness and Degree for VS (blue line), MCS (orange) and MCS/EMCS (green) clusters (N = 30, N = 32, N = 12, respectively), with the 13 variables on the y-axis and standardized (z-score) measures on the x axis (range − 2 to + 2). See Table 1 for key to variable names

**Fig. 9:**
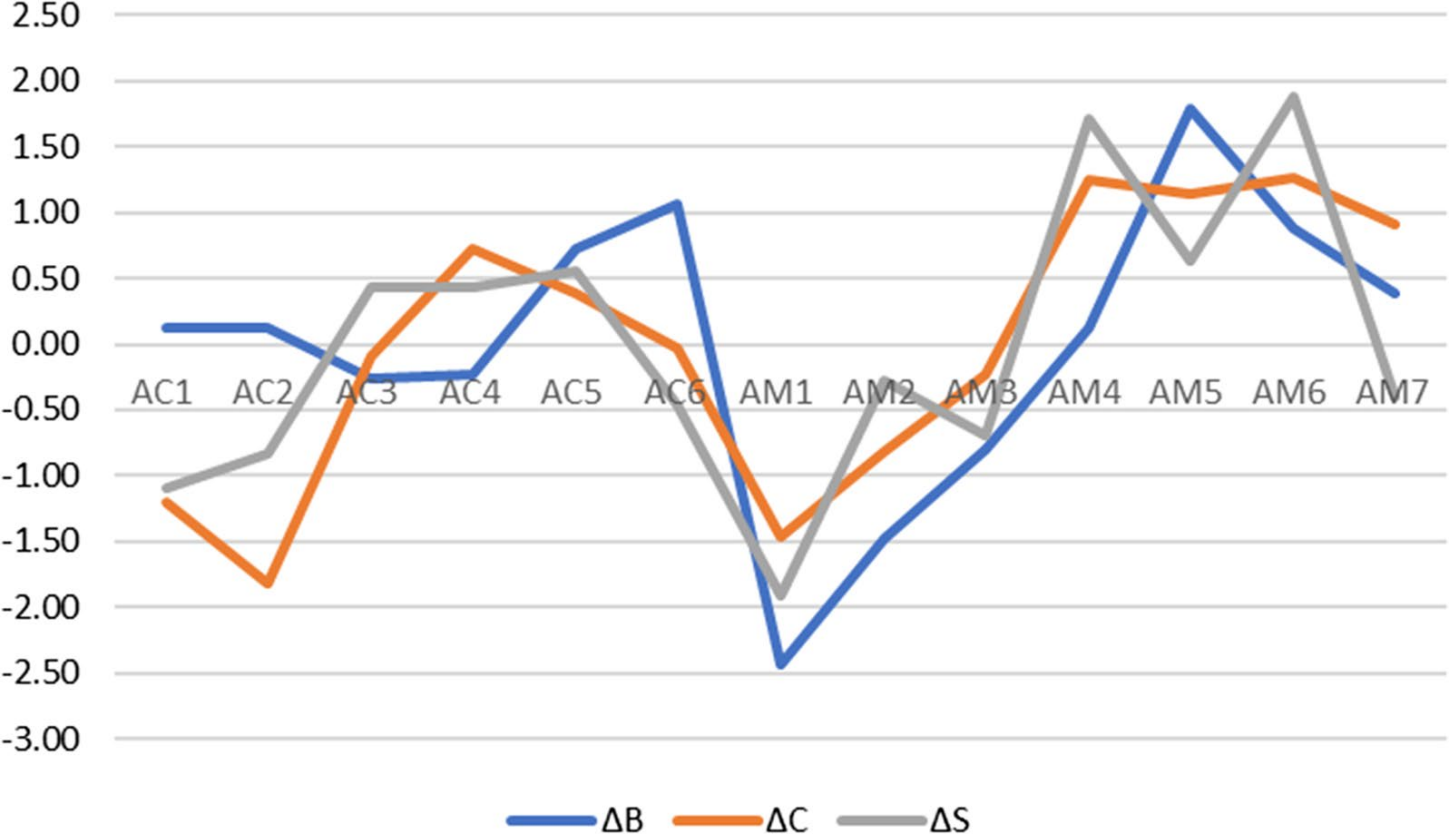
Graphical representation of the differences (∆s) in Betweenness (B), Closeness (C) and Strength (S) of induced categories VS and MCS (as given in the final three columns of Table 6). The y-axis goes from − 3 standard deviations to + 3 standard deviations (z scores). See Table 1 for key to variable names

Differences between the networks for induced category VS patients only (Fig. 5), for MCS patients only (Fig. 6) and for MCS/EMCS only (Fig. 7) are summarized through centrality plots (Fig. 8) and metrics (Table 6). The final three columns of Table 6 describe the difference between VS and MCS catgegories, as portrayed in Fig. 9.

**Table 6:**
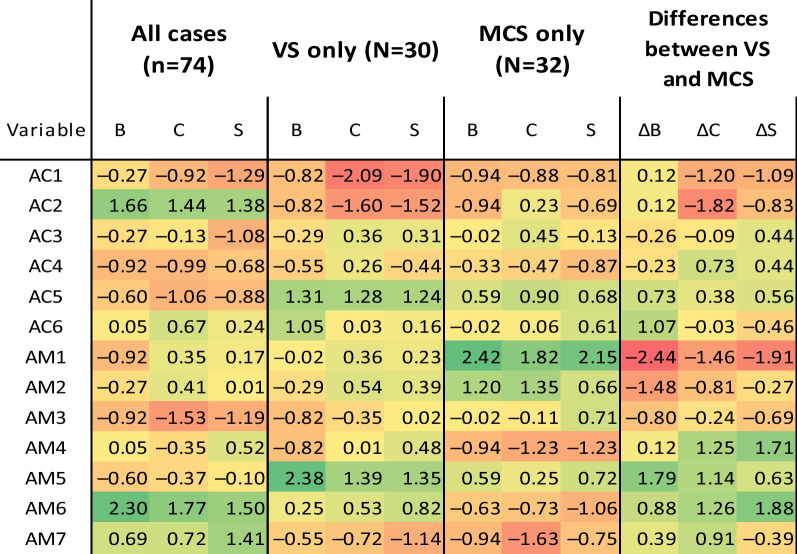
Summary of centrality metrics for all cases (Fig. 4), induced categories VS ( Fig. 5) and MCS (Fig. 6) (MCS/EMCS not included) for the 13 variables, with B = Betweenness, C = Closeness and S = Strength

The final three columns give the differences (∆s) between VS and MCS centrality figures. See Table 1 for key to variable names

## Discussion

For the exploratory objectives of this study, VS, MCS and EMCS were coded numerically as 0, 1 and 2, respectively, on the five items of the Principal MATADOC subscale. Original CRS-R subscale values were used for the six function scales. The four measurements taken of each patient were then averaged, as shown in Table 1, with most of the variables being reasonably symmetrical (skew − 0.5 to + 0.5) except for MVerbal, MMusical response and CAuditory (moderately skewed, − 1.0 to + 1.0), and CCommunication (highly skewed, greater than + 1). Negative kurtosis figures demonstrate mostly low to moderately flat distributions except for CCommunication (+ 5.80), which is peaked.

Analysis of variance shows significant relationships between individual variables and calculated diagnostic category for MATADOC and CRS-R (Fig. 1), leading to the possibility that ML may find interesting combinations of variables associated with these outcomes. McDonald’s ω was high for the MATADOC and CRS-R scales, indicating a good level of reliability for subsequent analysis. The high ω for the two scales combined provides evidence of concurrent validity of MATADOC with CRS-R. However, factor analysis (PCA) identified two separate structures in the combined scale, demonstrating lack of unidimensionality. Overall, these figures together with post-hoc power analysis provide assurance that the data are sufficiently reliable for subsequent exploration through machine learning and network analysis. The agreement between MATADOC and CRS-R calculated diagnostic outcomes is only fair, however, with 64% of cases having the same calculated outcome on both measures. This justifies the use of unsupervised ML to find common, induced categories for additional supervised ML and network analysis using all 13 variables.

A standard MLP neural network using all 13 variables produced a reasonably accurate (TPR 80%) model with cross-validation using MATADOC calculated outcome, and a slightly lower but significantly different 78% for CRS-R outcome (Tables 3(a), 4(a)). Including primary cause of injury (PCOI) as a factor reduced the accuracy of both to 77% and 74%, respectively (Table 4(a), middle), but improved sensitivity for VS from 20 cases to 22 for MATADOC, and from 29 cases to 30 for CRS-R (Table 3(a), middle). This indicates that PCOI may have a more predictive role in VS diagnosis than in MCS and EMCS.

PCOI was not needed when using J48, which returned 76% TPR rate for MATADOC and 95% for CRS-R (Tables 3(a) and 4(a), bottom). The latter figure may be explained by half the number of EMCS cases in the CRS-R diagnostic outcome in comparison to the MATADOC outcome (6 as opposed to 12, Table 4(a), bottom left and right), making it easier for the symbolic decision algorithm to find thresholds among the variables for identifying tests for a smaller number of cases. This was confirmed by the trees (Figs. 2, 3) found by J48, where PCOI was not needed. Data-fit models (no withheld cases) for MATADOC and CRS-R diagnostic outcomes both produced rule sets with 96% correct classification, with the MATADOC rule set using MATADOC items Visual Response, MATADOC Auditory Response and CRS-R Auditory Subscale (RS1). The CRS-R rule set used CRS-R scales Visual and Motor only (RS2). The inclusion of CRS-R Auditory as well as MATADOC Auditory Response in the rules for diagnosing MATADOC outcome (RS1) provides some evidence that these two variables measure different aspects of auditory response and complement each other in rule-based diagnosis.

Given the relatively low agreement between MATADOC and CRS-R diagnostic outcomes, k-means cluster analysis with the specified target of three clusters was used to identify three new induced categories of 30 cases for VS, 32 cases for MCS and 12 cases of EMCS/MCS, with cluster names identified by which group the original diagnostic outcome categories fell into. These induced categories improved the TPR performance of the cross-validated MLP significantly by 9% and 15% to 89% (Table 4(b), upper left) in comparison to the first MLPs that used MATADOC and CRS-R calculated outcomes (Table 4(a), upper left and right). This figure fell slightly to 87% if PCOI was added to the cross-validated MLP (Table 5(b), lower left), indicating that clustering may have found sufficient information in MATADOC and CRS-R data to partition cases into three groups without needing PCOI. The induced category performance for cross-validated J48 was 84% (Table 4(b), upper right), which is half-way between the original J48 performance of 76% and 95%, respectively, of the models using the originally calculated MATADOC and CRS-R outcomes (Table 4(a), bottom left and right). Adding PCOI did not affect J48 behavior.

Knowledge extraction from data-fitting MLP with induced categories identified MATADOC Behaviour (Behavioral Response to Music), MATADOC Musical Response and CRS-R Motor as the most important variables (Table 5). A data-fit J48 model with induced categories returned 99% accuracy and rule set with only one misclassified case (RS3). The three variables used were CRS-R Motor, MATADOC Verbal and MATADOC Musical response.

A partial correlation network analysis using all cases revealed that the two sets of variables had stronger relationships with each other than with variables in the other scale, but with some interesting cross-relationships (Fig. 4). MATADOC Behavioral Response to Music (AM6) and Musical Response (AM7) showed the strongest interlinks, as did MATADOC Awareness of Musical Stimuli (AM3) and Response to Verbal Commands (AM4). CRS-R Visual (AC2) was linked moderately strongly to CRS-R Motor (AC3) and Arousal (AC6). Also, cross-relationships between MATADOC Visual Response (AM1) and CRS-R Visual (AC2), and between MATADOC Arousal (AM5) and CRS-R Arousal (AC6), provide some evidence of convergent validity. Summed centrality measures (Table 6, columns 1–3) showed that MATADOC Behavioral Response to Music (AM6) had the most centrality (summed centrality of 5.57), followed by CRS-R Visual (AC2, 4.48) and MATADOC Musical Response (2.82).

The network for induced category VS (Fig. 5, Table 6 columns 4–6) showed major differences, with MATADOC Arousal (AM5) having the most summed centrality (5.12) followed by CRS-R Communication (AC5, 3.84). The network for MCS only (Fig. 6, columns 7–9) revealed that MATADOC Visual Response had the most centrality (6.39), followed by MATADOC Auditory Response (AM2). The biggest differences between centrality measures of VS and MCS were in MATADOC Visual Response (AM1, 4.31), MATADOC Auditory Response (AM2, 3.17) and CRS-R Visual (AC2, 3.06, Table 6 final three columns). Individual centrality differences between VS and MCS patients can be seen in Fig. 9. These networks show the importance of inducing new, combined categories from the data to allow all 13 variables to be interlinked, otherwise separate networks for each tool would have to be produced given the relatively low agreement in calculated outcomes.

A number of possible implications for clinical significance arise from these results. First, misdiagnosis of PDOC can be quite high (up to 40%) even by clinical consensus involving imaging [27], leading to continuing problems of intervention management [28]. Lack of agreement in diagnostic outcomes is evidenced in this study, with only fair agreement on MATADOC and CRS-R outcomes. ML performance showed improvement in PDOC diagnosis when diagnostic categories are based on both sets of data. Joining variables and outcomes in this way may point to data- and outcome-shared clinical decision-making strategies in future.

Second, a hybrid cluster emerged that contained MCS and EMCS cases from MATADOC and CRS-R variables, indicating that the transition from, and separation between, MCS to EMCS may need to be more carefully measured in future versions of these two tools. In particular, the induction of the hybrid cluster may provide evidence in support for an MCS + category [29]. Although used increasingly in clinical practice, MCS + is yet to gain wider acceptance and application in the PDOC research literature. The rules of RS3 for including patients in this hybrid cluster use MATADOC items Response to Verbal Commands and Musical Response, which may warrant examination as possible music-therapy variables for identifying a future MATADOC-based MCS + between MCS and EMCS.

Third, previous ML approaches have mostly involved ANNs [30], support vector machines (SVMs) [31], naïve Bayes (NB) [32], XGBoost (a form of ensemble method using gradient boosting [33]) and random forests [34] to predict mortality and poor outcome with a range of 60% to 80% accuracy. Differences in the way that model predictions are calculated as well as in the categories predicted make it difficult to compare the results presented here with previous results. However, the accuracy figures in this study show that PDOC diagnostic state may be predictable with at least the same accuracy as for mortality. This has implications for the design and development of future clinical decision-making tools for making predictions concerning diagnostic state and management of patients in different states in addition to long-term prognosis concerning mortality.

Furthermore, recent attempts to predict recovery (rather than mortality) with a variety of statistical and ML approaches using linear regression, SVMs, k-nearest neighbour, NB, decision trees and an ensemble approach tend towards a range of between 45 and 85% accuracy, depending on the type of cross-validation used, with leave-one-out performing better than tenfold [35]. Also, the data used in most previous studies varied greatly and involved demographic, clinical, radiographic and laboratory sources (e.g. [36, 37]). One of the problems in using a wide variety of data is that prior feature selection becomes important to prevent ML models from becoming saturated with too much irrelevant and redundant data and possibly not converging to an optimal or even sub-optimal point. But such feature selection, while common in ML, can become a problem area in its own right, since separating such irrelevant features can be computationally expensive [38]. The study reported here has used ANNs and decision trees with only 13 variables that have been previously tested to reveal relatedness in a number of validity studies. All 13 variables were obtained in situ by clinicians observing patients without the need to harvest data from other sources. ML, using these 13 variables, has managed to attain at least the same as, and in some cases better, accuracy than other models with much larger feature sets. In other words, this study has shown that, from a clinical diagnosis perspective, there may be sufficient information in a small number of narrowly focused and clinically-relevant neurobehavioral features for reasonably accurate diagnosis and prediction of PDOC state without the need for data harvested from multiple sources.

Also, unlike many of the previous ML approaches, the results reported here identify and extract knowledge concerning reasons for classification and prediction. For instance, RS1 predicted MATADOC outcome using MATADOC-specific Visual, Auditory and Verbal items together with Auditory function scale from CRS-R. RS2 predicted CRS-R outcome using just two function scales specific to CRS-R: Visual and Motor. RS3 predicted induced category based on Response to Verbal Command and Musical Response items from MATADOC, and the Motor function scale from CRS-R. These relationships can now be subject to further clinical investigation through targeted research to test their potential applicability as treatment regimes.

Lastly, our models not only identify the most important attributes on which predictions are made (in the case of ANNs by sensitivity analysis) and the reasons for predictions in the form of rules (in the case of J48), but also present differences between VS and MCS through network analysis. Such differences may allow possible transition pathways between VS and MCS to be identified using neurobehavioral measures and scales which are then open for critical evaluation in terms of clinical plausibility and reliability. This has not always been possible with previous application of ML in this area, which focused more narrowly on mortality and outcome using much wider sources of data. Figure 9 indicates that the centrality difference between VS and MCS is larger for MATADOC items than for CRS-R function scales, in particular, in MATADOC items Response to Verbal Commands, Arousal and Behavioral Response to Music. The clinical role of these three variables could be investigated in more detail to identify whether and how they could be potential targets for therapeutic intervention as part of transition management strategies to help patients move from VS to MCS.

## Conclusion

In summary, there has been a comparative lack of application of machine learning methods and techniques in Disorders of Consciousness studies in comparison to other clinical areas. The few pioneering studies that have been reported so far have used large sources of data in the search for critical markers of mortality and poor outcome. The approach in this study has been to use a narrow set of non-invasive measures including the criterion standard Coma Recovery Scale-Revised together with a more recently standardized music therapy measure MATADOC. This focus has allowed testing whether PDOC diagnosis could be aided by ML and whether network analysis could reveal differences between diagnostic states that can help in the identification of possible transition pathways for future clinical investigation. Using just 13 variables together with calculated and induced diagnostic states has led to models that, with cross-validation, are shown to be sufficiently accurate to warrant further investigation in clinical settings.

There are several limitations to this study, including limited sample numbers and measurements averaged over four occasions which may impact on the reliability as well as sensitivity of the analysis at specific time points. Also, it was not possible to test the models on totally new data not seen in model training and testing. Finally, no attempt was made to drill deeper into CRS-R function scales to identify the most important scale items within those scales. Nevertheless, the results presented here provide an early indication that music therapy can play a useful role in the diagnosis and management of PDOC cases, especially if it is used to supplement standard coma recovery strategies and methods. Also, some of the music therapy variables may have an important role in supporting new subcategories of diagnostic state which currently are specified by verbal and motor control behavior only.

## Supplementary Information

**Additional file 1.** Machine learning.

**Additional file 2.** Supplementary table.

## Data Availability

Data supporting the reported results are available upon reasonable request from the corresponding author.
