# Supplementary material for: Machine learning and network analysis for diagnosis and prediction in disorders of consciousness
Source: BMC Med Inform Decis Mak. 2023 Feb 28;23:41. doi: 10.1186/s12911-023-02128-0 (PMC9972731; doi:10.1186/s12911-023-02128-0)
Supplement: Supplementary file 1 — Additional file 1. Machine learning. [file 12911_2023_2128_MOESM1_ESM.docx]

**Additional file 1: Machine Learning (ML)**

The ANN perceptron for training and cross-validation consisted of 13 input units (one for every variable), 7 hidden units and two output units. The learning rate and momentum were 0.1, and the number or epochs (presentations of data) was 1000. This architecture as reached after a number of experiments and was found to be sufficient for 100% data fitting (i.e. no classification error when all samples were presented without any hold-out of cases). The transfer function at the hidden and output layers was sigmoid and the error function was steepest gradient descent. All cross-validation experiments were carried out using Weka (Waikato Environment for Knowledge Analysis developed at the University of Waikato in New Zealand), version 3.8.4.

Extraction of the most important variables for ANN classification was through SPSS 28.0.1.0. No testing was involved and the ANN architecture also contained 13 input units, 7 hidden units and 3 output units, with the same learning and momentum rates. The transfer functions were hyperbolic tangent at the hidden layer and softmax at the output layer (SPSS defaults). The error function was cross-entropy. Independent variable importance is calculated through sensitivity analysis using variance, where mean values of input nodes are adjusted to monitor effect on classification and normalized so that the most important variable is given 100% importance to identify relative importance of the remaining variables [1].

J48 is a popular decision tree algorithm for classification and prediction based on the C4.5 algorithm [2], and uses information entropy to generate models. The tree is constructed level by level by finding the most effective attribute test for splitting samples into as homogenous subsets as possible at the next level. The splitting criterion uses an information gain metrics based on entropy. Splitting stops when the number of samples in subsets drops below a certain number of the gain in information gain drops below a threshold. Rules can be generated from the tree by tracing all paths from the root node to the leaf nodes, with each path represented by a rule. Default values in Weka were used (minimum number of cases in a lead node = 2, confidence factor 0.25 for pruning trees). No reduced-error pruning was requested, non-binary splits were allowed, actual data value splits were required, a minimum of two cases per leaf was specified, parts were removed if they did not reduce training error, and data were randomly shuffled.

ML model construction consists of two stages (with variants): training and testing. During training, a certain proportion of the samples (training set) are used to identify a model to a certain specified degree of classification accuracy. The remaining samples (withheld test set) are then input with no further adjustment to the model to check the model output against the known class of the withheld samples. Variations (not used here) include a validation set (a small number of training samples used to optimize the initial trained model) and testing the model on totally new samples not in the initial training and test sets. It is normal in ML to refer to training as classification and testing as prediction.

Several methods exist for testing, or cross-validating, a trained model. Leave-one-out cross-validation builds a training model for all samples except one, and then tests the model against that one. This is repeated for every sample in the dataset. 10-fold cross-validation, as used in this study, splits the samples into ten equal-sized subsets. A model is then trained on nine of the subsets and tested on the tenth. This is repeated ten times and the results of the ten test sets reported.

Outcomes of machine learning for classification and prediction are reported in confusion matrices, which are tables where rows represent actual class of samples and columns the class predicted by the tested model. The leading diagonal of such tables identifies the number of samples correctly predicted (true positives), and the cells off the diagonal identify how errors have arisen. Other row cells identify false negative (FN) and other column cells false positives (FP). Three metrics are generated from confusion matrices: True positive rate (TPR, or sensitivity: true positives/(true positives + false negatives)), precision (P: true positives/(true positives + false positives)) and receiver operating characteristic area under the curve (ROC AUC: calculated by plotting TPR against false positive rate FPR at various threshold settings, where FPR=false positives/(false positives + true negatives)). A ROC (receiver operating characteristic) curve is a graphical plot of TP against the false positive rate at various threshold settings. The area under the curve (AUC) is the probability that a classifier will rank a randomly chosen positive samples higher than a randomly chosen negative sample.

K-means clustering is an supervised ML method in that the labels (class values) of samples are not used for model construction. Samples are partitioned into k-specified subsets (clusters) whereby, in principle, samples belonging to a cluster have more in common with each other than they do to samples belonging to another cluster. To do this, clustering requires measures: one for calculating the distance between samples, and the other for calculating differences between clusters. Euclidean distance using all the variables is used as the difference measure, and difference in means for calculating cluster centers and hence difference between clusters. K was set to 3 in this study.

**References**

[1] Saltelli A, Annoni P, Azzini I, Campolongo F, Ratto M, Tarantola S: **Variance based sensitivity analysis of model output. Design and estimator for the total sensitivity index**. *Computer Physics Communications* 2010, **181**(2):259-270.

[2] Salzberg SL: **C4.5: Programs for Machine Learning by J. Ross Quinlan. Morgan Kaufmann Publishers, Inc., 1993**. *Machine Learning* 1994, **16**(3):235-240.
