# Supplementary material for: Machine learning and network analysis for diagnosis and prediction in disorders of consciousness
Source: BMC Med Inform Decis Mak. 2023 Feb 28;23:41. doi: 10.1186/s12911-023-02128-0 (PMC9972731; doi:10.1186/s12911-023-02128-0)
Supplement: Supplementary file 2 — Additional file 2. Supplementary table. [file 12911_2023_2128_MOESM2_ESM.docx]

**Additional file 2**

*Supplementary Table: Breakdown of 13 variables and two scales by MATADOC and CRS-R outcomes*
